# Effects of burnout syndrome on pregnancy and the postpartum period

**DOI:** 10.1590/1806-9282.20250406

**Published:** 2025-12-05

**Authors:** Sezin Ateş

**Affiliations:** 1 Alanya Alaaddin Keykubat University, Faculty of Medicine, Department of Obstetrics and Gynecology – Alanya, Turkey.

**Keywords:** Pregnancy, Burnout, Postpartum, Breastfeeding

## Abstract

**OBJECTIVE:**

The aim of this study was to examine the prevalence of burnout among pregnant working individuals and its effects on both obstetric complications and issues that may arise in the early postpartum period.

**METHODS:**

Healthy pregnant women were questioned regarding their working life and Maslach Burnout Inventory. They were divided into two groups—burnout and non-burnout—according to the burnout score. The two groups were compared in terms of pregnancy and postpartum complications and breastfeeding.

**RESULTS:**

Burnout was identified in 164 pregnant women (54.1%). The age of the women with burnout was statistically significantly higher than that of the group without burnout. The breastfeeding rate was lower in the burnout group. In the evaluation of postpartum infective complications, 19.5% of the women with burnout and 8.6% of those without burnout developed infections, and this difference was statistically significant.

**CONCLUSION:**

It has been demonstrated that burnout increases the risk of infectious complications in pregnancy, reduces postpartum breastfeeding rates, and contributes to other negative outcomes.

## INTRODUCTION

Burnout syndrome is one of the most significant occupational psychosocial problems of our time and has become a major problem in terms of personal performance and financial costs for both individuals and organizations^1^. The concept of burnout was first defined by Freudenberger in 1974 as a response to stress resulting from prolonged exposure to adverse working conditions and occupational stress^2^. Although burnout syndrome was initially described as a problem specific to professions involving caregiving, subsequent studies and evidence have demonstrated that burnout can be observed across nearly all occupational groups^3^. Maslach identified exhaustion, fatigue, desperation, and hopelessness as the primary symptoms of burnout syndrome, which has been reported to be more common in professions that involve face-to-face interactions with people^3,4^.

The most widely used model is the burnout model developed by Maslach^3^. It consists of three distinct subdimensions: emotional exhaustion, depersonalization, and reduced personal accomplishment^5^. The emotional exhaustion dimension of burnout manifests as a feeling of depletion and fatigue resulting from the psychological effort exerted at work. The desensitization dimension is defined as an indifferent and unresponsive attitude toward the work itself and/or the people receiving the service. The reduced personal accomplishment dimension of burnout results in decreased productivity and skills, low morale, and diminished coping abilities^4^. The Maslach Burnout Inventory (MBI)^3^, defined by Maslach, is considered the gold standard for assessing the severity and risk of burnout^5^.

In this study, we aimed to examine the prevalence of burnout among pregnant working individuals and its effects on both obstetric complications and issues that may arise in the early postpartum period.

## METHODS

This study was conducted between April 2024 and January 2025 on pregnant individuals who presented to the Obstetrics and Gynecology Clinic of Alanya Training and Research Hospital. All pregnant individuals who presented at any stage of pregnancy were included in the study, and they were followed for 1 month in the postpartum period regarding infectious complications and breastfeeding status. Pregnant individuals over the age of 35 and those classified as high risk for obstetric complications due to various reasons were excluded from the study. The participants were administered the MBI, and data on occupation, duration of employment, working conditions, and sociodemographic characteristics were collected. Delivery mode, gestational age at birth, neonatal length and weight, obstetric complications, need for neonatal intensive care, postpartum infections, and breastfeeding status were recorded.

### Maslach Burnout Inventory and its assessment

In this study, the MBI, defined by Maslach and Jackson in 1981, was administered. This inventory consists of 22 items and evaluates burnout across three subdimensions: Emotional Exhaustion, Desensitization (Depersonalization), and Personal Accomplishment^3^. The Emotional Exhaustion subscale includes eight items assessing fatigue, weariness, and decreased emotional energy. Items 1, 2, 3, 6, 8, 13, 16, and 20 measure this dimension. The Desensitization subscale consists of six items evaluating an individual's emotionally detached behavior toward those they care for and serve (items 5, 10, 11, 15, 21, 22). The Reduced Personal Accomplishment subscale measures an individual's perception of their competence and success at work and comprises eight items (items 4, 7, 9, 12, 14, 17, 18, 19).

Participants were administered the Turkish-adapted version of the MBI, which was validated in 1992^6^. In this adaptation, the original seven-point response scale ("never, a few times a year, once a month, a few times a month, once a week, a few times a week, every day") was modified to a five-point Likert scale ("never, very rarely, sometimes, often, always").

### Scoring of the Maslach Burnout Inventory

The scores for the Emotional Exhaustion and Desensitization subscales were obtained by assigning 1 point for "never" and 5 points for "always," using a five-point Likert scale. Consistent with previous studies, in our study, burnout was considered to increase as scores in the Emotional Exhaustion and Desensitization subgroups rose and as scores in the Personal Accomplishment subgroup decreased^7^. The cutoff points were determined by subtracting the minimum possible score from the maximum and dividing the resulting value into three equal parts. The validity and reliability analyses of the five-point Likert version (1–5 points) of the MBI, adapted to Turkish and used in this study, reported Cronbach's alpha coefficients ranging between 0.60 and 0.83 (0.82–0.83 for Emotional Exhaustion, 0.60–0.71 for Desensitization, and 0.72–0.73 for Personal Accomplishment)^7^.

Based on this scoring system, scores of 30 and above in the Emotional Exhaustion subscale, all pregnant women were covered for their working life and were insured; 23 and above in the Desensitization subscale; and 8–18 in the Personal Accomplishment subscale were considered indicative of burnout^6^.

This study was approved by the Ethics Committee of Alanya Alaaddin Keykubat University Faculty of Medicine (Approval number: 19-09).

Exclusion criteria: Pregnant women over the age of 35; those with a history of thyroid disease, chronic hypertension, or diabetes before pregnancy those with recurrent miscarriage, preterm birth; those who use tobacco, alcohol, or substances; those with consanguineous marriage; and those with detected anomalies in previous pregnancies were excluded from the study.

Statistical analysis: Descriptive statistics of the data were presented as mean, standard deviation, median, minimum, maximum, frequency, and percentage values. The distribution of variables was assessed using the Kolmogorov-Smirnov and Shapiro-Wilk tests. The Mann-Whitney U test was used for the analysis of non-normally distributed independent quantitative variables, while the chi-square test was used for the analysis of independent categorical variables. All statistical analyses were performed using SPSS 28.0 software.

## RESULTS

A total of 337 pregnant women responded to the survey; however, 34 participants were excluded due to incomplete survey data or unanswered questions. Thus, statistical analysis was conducted on a total of 303 pregnant women. The mean age of the participants was 27 years (27.8±4.7). The average total work experience was calculated as 3 years. Among the participants, 170 pregnant women (56.1%) were employed in various sectors within the public and private sectors, while 133 pregnant women (43.9%) were self-employed. Regarding work schedule distribution, 97% (n=294) of the participants worked day shifts, while 3% (n=9) worked rotating shifts, including both day and night shifts. The sociodemographic characteristics, burnout levels, and pregnancy-related complications of the participants are presented in Table 1.

**Table 1:** Sociodemographic, burnout, and complication data of participants.

		Min–Max			Median	Mean±SD/n-%		
Age		17.0	–	40.0	27.0	27.8	±	4.7
Education level	Primary school					14		4.6%
	Middle school					44		14.5%
	High school					126		41.6%
	University					119		39.3%
Occupation	Private/public employee					170		56.1%
	Self-employed					133		43.9%
Work type	Day					294		97.0%
	Day–night					9		3.0%
Work hours		0.5	–	20.0	3.0	4.3	±	3.5
Gravida		1.0	–	6.0	2.0	1.9	±	1.0
Parity	(-)					160		52.8%
	(+)					143		47.2%
Parity number		1.0	–	4.0	1.0	1.5	±	0.6
Gestational week		5.0	–	41.0	34.0	31.3	±	8.1
SGA	(-)					264		87.1%
	(+)					39		12.9%
Oligohydramnios	(-)					280		92.4%
	(+)					23		7.6%
Delivery type	Vaginal					86		28.4%
	Cesarean					217		71.6%
Gestational week		28.0	–	41.0	39.0	38.7	±	1.4
Newborn weight		1,815	–	4,550	3,260	3,247	±	400
Need for intensive care unit	(-)					284		93.7%
	(+)					19		6.3%
Infection	(-)					273		90.1%
	(+)					44		14,5%
Breastfeeding status	(-)					97		32.0%
	(+)					206		68.0%
Emotional exhaustion score		9.0	–	44.0	20.0	20.8	±	7.2
Depersonalization score		5.0	–	20.0	8.0	8.8	±	3.6
Reduced personal accomplishment score		8.0	–	40.0	29.0	27.5	±	7.9
Burnout	(-)					139		45.9%
	(+)					164		54.1%

SGA: small for gestational age; SD: standard deviation.

In the study, burnout was identified in 164 pregnant women (54.1%). The age of the women with burnout was statistically significantly higher than that of the group without burnout (p<0.05). In the comparison of obstetric complications, no significant differences were observed between the groups with and without burnout in terms of SGA, oligohydramnios rates, mode of delivery, gestational week, newborn weight, newborn length, and ICU admission (p>0.05). In the study, the breastfeeding rate was 64% in the women with burnout and 72.7% in those without burnout, with this difference being statistically significant (p<0.05). A total of 44 patients (14.5%) developed infectious complications (mastitis, wound infection). In the evaluation of postpartum infective complications, 19.5% (n: 32) of the women with burnout and 8.6% (n: 12) of those without burnout developed infections, and this difference was statistically significant (p<0.05). The comparison data of women with and without burnout are presented in Table 2.

**Table 2:** Comparison of sociodemographic data and complications on pregnant women who were seen but not evaluated for burnout.

		Burnout (-) (n: 139)				Burnout (+) (n: 164)				p-value
		Mean±ss/n-%			Median	Mean±ss/n-%			Median	
Age		27.2	±	4.9	26.0	28.3	±	4.5	28.0	***0.009*** m
Education level	Primary school	4		2.9%		10		6.1%		0.397c²
	Middle school	20		14.4%		24		14.6%		
	High school	64		46.0%		62		37.8%		
	University	51		36.7%		68		41.5%		
Occupation	Private/public	83		59.7%		87		53.0%		0.244c²
	Self-employed	17		12.2%		15		9.1%		
Work type	Day	136		97.8%		158		96.3%		0.443c²
	Day–night	3		2.2%		6		3.7%		
Work hours		3.99	±	3.40	3.00	4.54	±	3.64	4.00	0.162^m^
Gravida		1.86	±	1.04	1.00	1.94	±	1.04	2.00	0.406^m^
Parity	(-)	77		55.4%		83		50.6%		0.406c²
	(+)	62		44.6%		81		49.4%		
Parity number		1.50	±	0.59	1.00	1.43	±	0.67	1.00	0.292^m^
Gestational week		32.6	±	6.7	34.0	30.3	±	9.0	34.0	0.115^m^
SGA	(-)	123		88.5%		141		86.0%		0.515c²
	(+)	16		11.5%		23		14.0%		
Oligohydramnios	(-)	128		92.1%		152		92.7%		0.845c²
	(+)	11		7.9%		12		7.3%		
Delivery type	Vaginal	38		27.3%		48		29.3%		0.710c²
	Cesarean	101		72.7%		116		70.7%		
Gestational week		38.8	±	1.5	39.0	38.7	±	1.4	39.0	0.208^m^
Newborn weight		3,261	±	428	3,300	3,236	±	375	3,215	0.268^m^
Need for intensive care unit	(-)	129		92.8%		155		94.5%		0.542c²
	(+)	10		7.2%		9		5.5%		
Infection	(-)	127		91.4%		132		80.5%		***0.007*** c²
	(+)	12		8.6%		32		19.5%		
Breastfeeding status	(-)	38		27.3%		59		36.0%		***0.006*** c²
	(+)	101		72.7%		105		64.0%		
Emotional exhaustion score		15.5	±	4.8	15.0	25.2	±	5.8	25.0	***0.000*** m
Depersonalization score		6.1	±	1.7	5.0	11.1	±	3.3	10.0	***0.000*** m
Reduced personal accomplishment score		27.5	±	9.2	30.0	27.5	±	6.6	29.0	0.355^m^

SGA: small for gestational age;

m Mann-Whitney U test;

c² Chi-square test.

Significant p-values are indicated in bold and italic. p<0.05 values (statistically valuable).

## DISCUSSION

The prevalence of burnout syndrome varies significantly across different professions and countries. Even within the same country and profession, the prevalence can differ significantly. For example, a national study conducted in the United States reported burnout prevalence among surgical residents ranging from 3.2 to 91.4%, with 43.2% of individuals exhibiting weekly burnout symptoms^8^. The factors contributing to the development of burnout can primarily be categorized into individual-related and work-related factors^9^. Particularly, poor working hours, a negative and overly controlling employer environment, role and task ambiguity in the workplace, lack of social support, lack of autonomy in the workplace, and a highly intense and stressful working environment are considered the main job-related factors that contribute to the development of burnout^10^.

Regarding individual factors that contribute to burnout development, some studies have shown that burnout decreases as age increases^11^, while others have reported that the component of personal accomplishment in burnout declines with age^12^.

In our study, the burnout prevalence among pregnant workers across various fields was found to be 54.1%. Although no studies on burnout prevalence in pregnant women were found in the English literature to allow for a direct comparison, the fact that more than half of the pregnant individuals in our study experienced burnout is a noteworthy finding. In a previous study, the prevalence of burnout during the postpartum period within 1 year was reported to be 10%^13^.

Since our study focused on pregnant workers, burnout risks in other occupational groups were not analyzed; however, the participants were divided into two groups: those working in their own workplace and those working in other people's workplaces (private or public sectors). No statistically significant difference in burnout prevalence was observed between these two groups. When examined by work schedule, no significant difference in burnout prevalence was found between daytime workers and those working both day and night shifts (rotational). Similarly, no significant difference in burnout prevalence was observed in relation to the duration of employment. In our study, although pregnant women over the age of 35 were excluded from the analysis due to increased pregnancy complications, age-related analysis revealed that the prevalence of burnout increased with age. This is important because it suggests that the risk of burnout and the associated complications during pregnancy and the postpartum period may increase in advanced maternal age pregnancies, which are known to have higher obstetric risks. When the pregnant participants were included in the study, the gestational week was recorded. The pregnant groups with and without burnout were compared in terms of gestational week; no statistically significant difference was found between the two groups. Accordingly to these results, the frequency of burnout during pregnancy is not affected by gestational week.

Studies have also shown that, alongside its psychological effects, burnout syndrome can lead to numerous physical health problems. It has been associated with cardiovascular diseases, type 2 diabetes, obesity, hypercholesterolemia, coronary artery disease, gastrointestinal issues, respiratory infections, severe injuries, insomnia, musculoskeletal problems, and headaches^14^. In our study, the negative effects of burnout syndrome on pregnancy and the postpartum period in working pregnant women were investigated. When comparing pregnant women with and without burnout, no significant differences were found between the groups in terms of SGA, oligohydramnios, mode of delivery, gestational age, newborn weight, newborn length, and newborn ICU admission rates.

There are studies showing that burnout syndrome increases the risk of infectious problems. In individuals with burnout syndrome, it has been reported that the risk of developing conditions such as the common cold, flu, and gastroenteritis increases due to insufficient immune functions^15^. Another study examined the effect of burnout syndrome on infectious problems and found that individuals with a history of four or more infectious diseases and hospitalizations due to infectious diseases in the last year had higher rates of emotional exhaustion and depersonalization compared to individuals without a history of infectious diseases in the past year^16^. In our study, the incidence of infectious complications such as wound infections and mastitis was 19.5% in pregnant women with burnout, while it was 8.6% in those without burnout. This difference was found to be statistically significant. The higher incidence of infections in patients with burnout may be attributed not only to immune insufficiency but also to neglecting personal care tasks such as wound and breast care. Furthermore, burnout syndrome's contribution to increased infectious complications also leads to longer antibiotic use, which, in turn, results in increased treatment costs and indirect negative effects on bacterial resistance.

Numerous studies have reported factors that negatively affect breastfeeding and its duration in the postpartum period. It has been reported that depression has negative effects on breastfeeding. In one study, postpartum depression occurring in the first 4–6 weeks after childbirth was found to extend up to 1 year, with the most significant impact during the first 6 months. The study also reported that depressed mothers feel dissatisfaction during breastfeeding and feel more vulnerable to discontinuing breastfeeding^17^. In this study, we found that the rate of breastfeeding in the postpartum period was statistically significantly lower in pregnant women with burnout compared to those without burnout (64 vs. 72.7%). Considering the number of women who developed mastitis in our study, it is not possible to attribute the lower breastfeeding rate solely to the development of mastitis. Moreover, since mastitis is not contraindicated for breastfeeding, and given that the risk of mastitis and wound infections is higher in pregnant women with burnout, it can be concluded that burnout syndrome presents a significant risk for postpartum complications in pregnant women.

In conclusion, the study examining the frequency of burnout and its potential consequences in pregnant women working in various fields revealed that the incidence of burnout in pregnant women can be high. Undoubtedly, further studies on the subject are needed, particularly among working pregnant women. However, based on the data obtained in our study, it has been demonstrated that burnout increases the risk of infectious complications in pregnancy, reduces postpartum breastfeeding rates, and contributes to other negative outcomes. From the perspective of maternal and infant health, it is crucial to consider burnout syndrome in working pregnant women and initiate psychosocial support and preventive management when it is detected.

Limitations of the study: The present study has some limitations, and knowing these limitations will guide future researchers. First, data were collected from only one center, so the number of participants in the study is limited. Future studies can be planned as multicentered and conducted with a larger number of participants. Second, according to our review, there is no study on the subject in English. Therefore, we were able to compare our results with nonpregnancy studies.

## Data Availability

The datasets generated and/or analyzed during the current study are available from the corresponding author upon reasonable request.
